# Acquired Phototrophy through Retention of Functional Chloroplasts Increases Growth Efficiency of the Sea Slug *Elysia viridis*

**DOI:** 10.1371/journal.pone.0120874

**Published:** 2015-04-01

**Authors:** Finn A. Baumgartner, Henrik Pavia, Gunilla B. Toth

**Affiliations:** University of Gothenburg, Department of Biological and Environmental Sciences-Tjärnö, Strömstad, Sweden; College of Charleston, UNITED STATES

## Abstract

Photosynthesis is a fundamental process sustaining heterotrophic organisms at all trophic levels. Some mixotrophs can retain functional chloroplasts from food (kleptoplasty), and it is hypothesized that carbon acquired through kleptoplasty may enhance trophic energy transfer through increased host growth efficiency. Sacoglossan sea slugs are the only known metazoans capable of kleptoplasty, but the relative fitness contributions of heterotrophy through grazing, and phototrophy via kleptoplasts, are not well understood. Fitness benefits (i.e. increased survival or growth) of kleptoplasty in sacoglossans are commonly studied in ecologically unrealistic conditions under extended periods of complete darkness and/or starvation. We compared the growth efficiency of the sacoglossan *Elysia viridis* with access to algal diets providing kleptoplasts of differing functionality under ecologically relevant light conditions. Individuals fed *Codium fragile*, which provide highly functional kleptoplasts, nearly doubled their growth efficiency under high compared to low light. In contrast, individuals fed *Cladophora rupestris*, which provided kleptoplasts of limited functionality, showed no difference in growth efficiency between light treatments. Slugs feeding on *Codium*, but not on *Cladophora*, showed higher relative electron transport rates (rETR) in high compared to low light. Furthermore, there were no differences in the consumption rates of the slugs between different light treatments, and only small differences in nutritional traits of algal diets, indicating that the increased growth efficiency of *E*. *viridis* feeding on *Codium* was due to retention of functional kleptoplasts. Our results show that functional kleptoplasts from *Codium* can provide sacoglossan sea slugs with fitness advantages through photosynthesis.

## Introduction

Photosynthesis by plants, algae, and some bacteria is a fundamental process that provides oxygen and energy that sustains heterotrophic life. However, several mixotrophic organisms are able to obtain organic carbon via both heterotrophic and phototrophic metabolism through acquired phototrophy (AcPh) [1–4]. AcPh includes both the capability of harbouring photosynthetic algae or bacteria as independent entities (endosymbiosis) [4] and the retention of viable chloroplasts (kleptoplasty) [5]. Photosynthesis in eukaryotes is hypothesized to have evolved through endosymbiotic events resembling AcPh [5], and therefore, the physiology, as well as cellular and genetic mechanisms of AcPhs has been widely studied [2,4,6]. AcPh likely leads to increased ecosystem production through efficient recycling of metabolites resulting in increased gross growth efficiency of the host [3]. However, a full understanding of the ecological and evolutionary benefits of AcPh is not clear and basic research, especially on kleptoplasty, is lacking for many species [2,3].

Kleptoplasty is a widespread phenomenon in protists [2,3], but is only known from one taxon of metazoans, the Sacoglossa (Mollusca, Opisthobranchia) [7]. The mechanisms that allow sacoglossans to retain ingested chloroplasts in a functional state over weeks to months of starvation are relatively well studied [4,8–10], but not well understood, as is also the case for the ecological significance of kleptoplasty [11]. Fitness benefits (i.e. increased survival or growth) of kleptoplasty in sacoglossans are commonly studied under extended periods of complete darkness and/or starvation ([12–16] but see [17] where photosynthesis inhibitors were used). Kleptoplasts can provide sacoglossans with photosynthates during periods of food shortage, which serve to extend survival or reduce mass loss under light versus dark conditions [13,15,16,18], although this notion has recently been challenged [17,19]. Kleptoplast functionality under starvation varies from less than a few months in most sacoglossan species [12,20–22] to the entire life cycle (≈ 10 months) in *Elysia chlorotica* [18,23]. Short periods of kleptoplast functionality may be due to the fact that kleptoplasts, which often degrade under high light irradiances [24,25], are not replenished in starved individuals, and may be the reason why previous studies have failed to detect fitness benefits in the form of increased growth [13–16]. The relative fitness contributions of heterotrophy, through grazing, and phototrophy, via kleptoplasts, under ecologically relevant conditions are not well characterized among kleptoplastic sea slugs ([11,26,27] but see [28]).

*Elysia viridis*, at the population level, is a dietary generalist that associates with up to 8 different genera of macroalgae throughout its geographical range [29]. Algal food sources most commonly cited include species of the green algal genera *Codium*, *Cladophora*, *Chaetomorpha*, and *Bryopsis* [30]. However, most studies on kleptoplasty in this species have focussed on kleptoplasts from the macroalgal genus *Codium*. Kleptoplasts from this genus (predominantly *Codium fragile*) demonstrate substantial light dependent carbon fixation [31], and functionality (measured via pulse amplitude modulated [PAM] fluorometry) [24,32,33], and have been implicated in the assimilation of several forms of nitrogenous compounds in *E*. *viridis* [26]. Furthermore, *E*. *viridis* survival and reductions in mass loss in light under starvation have been attributed to the retention of kleptoplasts from *Codium*, which can remain functional for several months of starvation [12,13,24,32,34]. However, no fitness benefits (i.e. weight gain) attributable to photosynthesis by functional kleptoplasts in satiated *E*. *viridis* have been found previously [13].

The present study investigates the possible fitness benefits of functional kleptoplasts in *Elysia viridis* under ecologically relevant light and food conditions. Growth efficiency (GE) was measured in slugs fed two algal host species (*Codium fragile* and *Cladophora rupestris*, henceforth referred to as *Codium* and *Cladophora*) under two light regimes for four weeks. Furthermore, we studied if increases in GE were correlated to increased photosynthetic functionality of kleptoplasts in *E*. *viridis* (assessed as relative electron transport rate, rETR). Because *Codium* provides *E*. *viridis* with kleptoplasts of high functionality, we specifically hypothesized that *E*. *viridis* originating from, and maintained on, *Codium* hosts would show a higher GE and rETR in high compared to low light. Kleptoplasts from *Cladophora*, on the other hand, have limited functionality (F. A. Baumgartner, unpublished), and therefore, we specifically hypothesized that slugs fed on this diet would show a similar GE and rETR irrespective of the light regime. Furthermore, we hypothesized that if slugs receive a fitness benefit due to an increased rETR through functional kleptoplasts, the GE would be correlated to the rETR, but not to any nutritional traits, of the macroalgal diets.

## Materials and Methods

### *Elysia viridis* growth experiments

*E*. *viridis* were collected from the algal species *Codium* and *Cladophora* at two field sites in the Koster Fjord, Sweden (Yttre Vattenholmen 58° 52’ 33.5” N, 11°6’ 22.9” E and Saltö Lyngnholmen 58° 51’ 45.3” N, 11° 7’ 52.8”) in late July/early August 2011 and 2012 respectively. No specific permissions were required to sample organisms at these locations and the study did not involve any endangered or protected species. Slugs were kept in 5 L outdoor aquaria with running seawater and provided their original algal host *ad libitum* as food for one month. Three days prior to the growth experiments, *E*. *viridis* were transferred indoors to adapt to low light conditions (photosynthetic photon flux density [PPFD] of 4–8 μmol quanta. m^-2^. s^-1^). At the start of the experiments, 40 individuals were patted dry with absorbent paper, weighed (± 1 mg) and left overnight in separate 0.2 L aquaria with their original host algae as food. The following morning (2–3 h into day conditions) kleptoplast functionality (*F*_v_/*F*_m_, see below for explanation) of each *E*. *viridis* was measured and slugs were then placed in either a low (5–7 μmol quanta. m^-2^. s^-1^) or high (85–105 μmol. quanta. m^-2^. s^-1^) light treatment and provided their original algal host as food. Light was provided by fluorescent tubes (L36W/840 Active Daywhite, Osram, Germany) and the low light treatment was produced by covering tubes with neutral density mesh. After 28 days, *E*. *viridis* were reweighed and growth rate (GR) (mg d^-1^) was calculated as:

$$GR=\frac{\left(M_{E}-M_{B}\right)}{t}$$

where M_E_ is the mass at the end of the experiment, M_B_ the mass before the experiment, and t refers to time in days of the experiments. Algal diets were replaced with freshly collected specimens every week. Prior to their use in the experiments, algal diets were cleaned of organisms, placed in separate 5L indoor aquaria under a PPFD of 4–8 μmol quanta. m^-2^. s^-1^ for 24 h. Both experiments (i.e. *Codium* 2011 and *Cladophora* 2012) were conducted in a constant temperature room kept at 18.5 ± 1°C under a 12:12-h light:dark photoregime. Seawater (salinity 20.7 ± 0.9 ‰) was exchanged daily in all experimental units.

### *Elysia viridis* algal consumption rate

Once per week during experiments, algal consumption rate (CR) by *E*. *viridis* in both light treatments was assessed. *E*. *viridis* feeds by piercing and sucking cytoplasm from the algal cells, and because *Codium* and *Cladophora* have different morphologies (siphonous/coenocytic vs. filamentous/septate), consumption was assessed using slightly different methods. Because *Codium* does not have defined cell walls, consumption was based on algal mass change and was measured for each *E*. *viridis* individual each week. Consumption on *Cladophora*, on the other hand, was based on the number of damaged cells, and could only be assessed for five *E*. *viridis* individuals from each light treatment each week due to time constraints (i.e. the consumption rate of each *E*. *viridis* individual was assessed once during the *Cladophora* experiment). In order to control for autogenic changes not associated with feeding, genetically identical pieces of algal diets without *E*. *viridis* were prepared in separate aquaria and kept under both light treatments. The mass or number of damaged cells was determined prior to commencing an assay and when > 50% of the algal diet piece appeared damaged or after a maximum of 24 h (*Cladophora*) or 44 h (*Codium*). CR (mg d^-1^ for *Codium* and cells d^-1^ for *Cladophora*) by each individual *E*. *viridis* was calculated using the following equations:

$$CR=\frac{G_{B}\left(\frac{C_{E}}{C_{B}}\right)-G_{E}}{t}$$

$$CR=\frac{\left(G_{E}-C_{B}\right)-\left(C_{E}-C_{B}\right)}{t}$$

where G_B_ and C_B_ represent the grazed and control algal diets before, and G_E_ and C_E_ represent the grazed and control algal diets at the end of the consumption assay. t refers to time in days of the assay. After termination of a consumption experiment individuals were provided with excess food.

### *Elysia viridis* growth efficiency

Using measures of GR and CR, growth efficiency (GE) (mg g^-1^ algae consumed for *Codium* and mg 1000 algal cells^-1^ consumed for *Cladophora*) was calculated for each individual:

$$GE=\frac{GR_{l,i}}{meanCR_{l,i}}1000$$

$$GE=\frac{GR_{l,i}}{meanCR_{l}}1000$$

where the subscript i relates to the *E*. *viridis* individual (1–20) and l the light treatment (Low or High). For *Codium* experiments mean CR (mg d^-1^) during the experiment was determined for each *E*. *viridis* by calculating the average consumption rate for each individual over the entire experiment (n = 4 per individual). For *Cladophora* experiments mean CR (cells d^-1^) during the experiment were determined by calculating the average CR of *E*. *viridis* within each light treatment over the entire experiment (n = 20 per light treatment).

### Chlorophyll *a* fluorescence

To assess the health and functionality of kleptoplasts in *E*. *viridis* and chloroplasts in algal diets, the quantum yield of chlorophyll *a* fluorescence was measured using PAM fluorometry. This measure assesses the proportion of reaction centres (RCs) in photosystem II (PSII) capable of utilizing photosynthetic energy and can be measured after dark-adaptation, providing maximum quantum yield (*F*_v_/*F*_m_ = (*F*_m_- *F*_o_)/*F*_m_), or under ambient light conditions, providing the effective quantum yield (Δ*F*/*F*_m_’ = (*F*_m_’- *F*)/ *F*_m_’). Minimum fluorescence (*F*_o_) occurs when all PSII RCs are open/oxidised, a subsequent saturating pulse of light is then applied, which closes/reduces RCs, yielding the maximum fluorescence (*F*_m_). *F*_o_ and *F*_m_ are measured after a period of darkness when all photochemical processes are relaxed. *F* and *F*_m_’ are the equivalent of *F*_o_ and *F*_m_ but are measured under ambient light conditions [35].

Fluorescence measurements were carried out using a Diving-PAM (Walz GmbH, Effeltrich, Germany) equipped with a red measuring light (light emitting diode (LED, 650 nm), frequency = 0.6 KHz, 0.15 μmol quanta. m^-2^. s^-1^, measuring light intensity setting = 8), assessing *F*_*o*_ or *F*. Transition from *F*_*o*_ or *F* to *F*_m_ *or F*_m_’ was induced via a saturating flash (halogen, white light) of 0.6 s with a peak intensity of ~8000 μmol quanta. m^-2^. s^-1^ (saturation-intensity setting = 8). Measuring light and saturation pulses were provided to a sample via a fibreoptics bundle (5.5 mm working diameter) that was fixed 10 mm from the surface of a white tray using a 60° distance clip (Walz Gmbh, Effeltrich, Germany). Prior to assessing the quantum yield of either slugs or algae, any background signal was suppressed by applying the auto-zero function of the diving-PAM on the small hole in the 60° distance clip, filled with seawater, without a sample present. Samples were then carefully positioned in the centre of the hole, covered with seawater, and *F*_v_/*F*_m_ or Δ*F*/*F*_m_’ measured. Care was taken to ensure that samples were placed in the centre of the hole and that movement was minimal during measurements, as movement can cause significant deviations in assessments of quantum yield [24]. Small pieces of algal diets (tip for *Codium* or several filaments for *Cladophora*) were measured in a similar manner.

*F*_v_/*F*_m_ was used as a proxy to assess the health of PSII RCs in *E*. *viridis* and algal diets prior to their use in experiments and was measured after samples had been dark adapted for 15–30 min. Δ*F*/*F*_m_’ was used in conjunction with PPFD in each light treatment to estimate the rETR. rETR is a measurement of electron transport through the photo-chemical reactions leading to carbon fixation [36] and was calculated as rETR = Δ*F*/*F*_m_’ × PPFD [37]. Δ*F*/*F*_m_’ was measured at a PPFD of 6 μmol quanta. m^-2^. s^-1^ or 80 μmol quanta. m^-2^. s^-1^ for low and high light treatments respectively. Δ*F*/*F*_m_’ was assessed in all *E*. *viridis* and algal pieces at the end of each week (i.e. n = 4 repeated measures of *E*. *viridis* individuals and n = 1 for algal pieces).

### Algal photophysiology and nutritional content

In each week of an experiment, *F*_v_/*F*_m_ of two pieces of each collected algal individual (n = 5) were assessed, and then transferred to separate aquaria (0.2 L) with one piece placed in each of the light treatments alongside *E*. *viridis*. After one week in light treatments, Δ*F*/*F*_m_’ was assessed at 6 μmol quanta. m^-2.^ s^-1^ or 80 μmol quanta. m^-2.^ s^-1^ for pieces in low and high light treatments respectively and the rETR calculated (as above). Algal pieces were then patted dry, weighed and frozen (-80°C) until nutritional analyses. This process was repeated each week (n = 4) during an experiment.

For analysis of nitrogen (N), carbon (C), and protein content, algal pieces were freeze-dried (22 h), reweighed and ground to a powder with a Ball Mill (Retsch MM301). N and C content of algal pieces were measured by combustion (2.91–3.15 mg / algal piece) using a Fisons EA1108 CHNS-O element analyzer. Protein content of algal pieces were measured colourmetrically using a modified version of Bradford’s method [38]. Although this method does not provide the absolute amount of soluble protein it is considered a reliable measure to compare soluble protein content in algae [39].

### Statistical analysis

Data for *E*. *viridis* initial mass, *F*_v_/*F*_*m*_, CR, and GE were analysed by t-tests comparing the effect of different light treatments. When variances were heterogeneous t-tests using separate variance were used [40]. Data for rETR of *E*. *viridis* were analysed by a 2-factor repeated measures ANOVA with light treatment (2 levels) as the fixed between subjects factor and time (4 levels) as the fixed within subjects factor.

Data for algal protein, N, C, C:N, dry weight, *F*_v_/*F*_m_ and rETR were analysed separately for each algal diet species by 2-factor ANOVAs with light treatment (2 levels) and time (4 levels) as fixed, orthogonal factors. Data were log transformed when required to satisfy homogeneity of variance.

## Results

The data underlying the statistical analyses in the present study can be found in (S1 Table).

There was no significant difference in the mean initial mass and kleptoplast functionality (*F*_v_/*F*_*m*_) of *E*. *viridis* from *Codium* (*t*-test, initial mass: *t*_1,38_ = 0.153, *P* = 0.879; *F*_v_/*F*_*m*_: *t*_1,38_ = 0.672, *P* = 0.506) or *Cladophora* (*t*-test, initial mass: *t*_1,38_ = 0.076, *P* = 0.940; *F*_v_/*F*_*m*_: *t*_1,38_ = 0.075, *P* = 0.940) chosen for the different light treatments in the four-week growth experiments. The initial mass and *F*_v_/*F*_*m*_ of *E*. *viridis* from *Codium* exposed to low light was 18.75 ± 0.71 mg and 0.768 ± 0.005 (means ± SEM) and to high light was 18.60 ± 0.68 mg and 0.766 ± 0.004 (means ± SEM). The corresponding values for *E*. *viridis* from *Cladophora* were 17.95 ± 0.91 mg and 0.314 ± 0.025 (means ± SEM), and 17.85 ± 0.96 mg and 0.311 ± 0.022 (means ± SEM) for low and high light treatments respectively.

**Fig 1:**
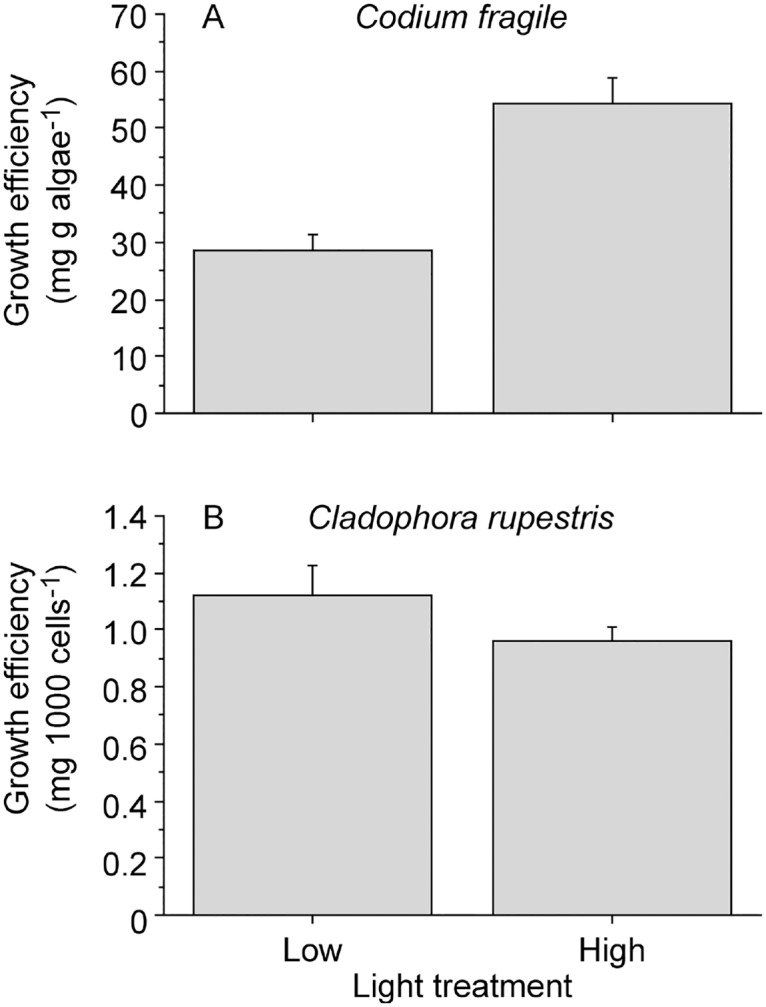
Growth efficiency of *Elysia viridis* from A) *Codium fragile* (mg per g algae) and B) *Cladophora rupestris* (mg per 1000 algal cells consumed) fed their original algal host diets under low and high light at the end of four-week experiments. Error bars show + SEM (n = 20).

There was a statistically significant difference in the GE of *E*. *viridis* from *Codium* at the end of the experiment (t-test, *t*_1,38_ = 4.965, *P* < 0.001). Slugs kept in the high light treatment grew almost twice as much per unit consumption as the slugs kept under low light (Fig. 1a), but there was no significant difference in the amount of *Codium* consumed by the slugs during the experiment (t-test, *t*_1,38_ = 0.678, *P* = 0.416). On average, slugs kept under low and high light consumed 54.43 ± 4.13 (mean ± SEM) and 49.34 ± 4.61 (mean ± SEM) mg of algal tissue per day respectively. In contrast, *E*. *viridis* from *Cladophora* did not differ significantly in their GE when exposed to different light treatments (t-test, *t*_1,38_ = 1.412, *P* = 0.166; Fig. 1b). Furthermore, slugs consumed a similar number of algal cells during the experiment (t-test, *t*_1,32_ = 3.230, *P* = 0.156). On average, slugs kept under low and high light consumed 810 ± 71 (mean ± SEM) and 971 ± 86 (mean ± SEM) algal cells per day respectively. These results indicate that *E*. *viridis* consuming *Codium*, but not slugs fed *Cladophora*, can gain a fitness benefit from functional kleptoplasts retained from their green algal hosts.

**Fig 2:**
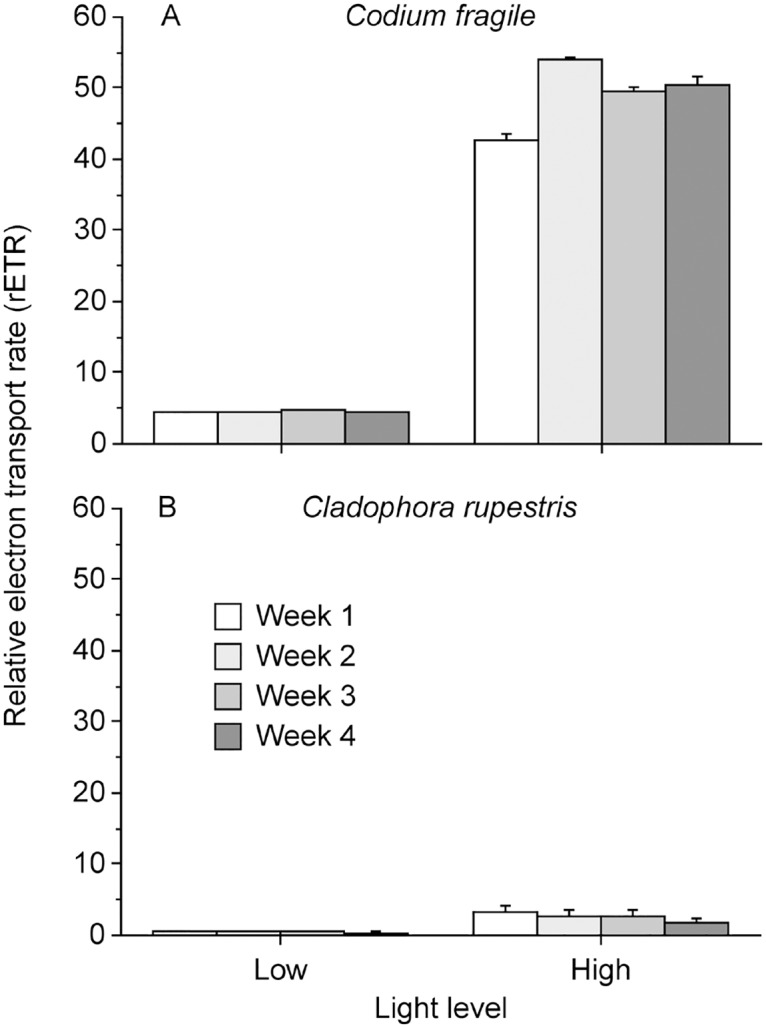
Relative electron transport rate of photosystem II (rETR) in *Elysia viridis* from A) *Codium fragile* and B) *Cladophora rupestris* fed their original algal host diets under low and high light during four-week experiments. Error bars show + SEM (n = 20).

There was significant temporal variation in the rETR of *E*. *viridis* consuming *Codium*, but not *Cladophora*, during the four-week experiments, and the rETR also differed significantly between slugs in the low and high light treatments (Table 1a,b, Fig. 2a,b). However, variances around means were very small and although statistically significant, the difference in rETR among different sampling occasions within light treatments for both types of slugs, as well as between light treatments for *Cladophora* slugs, was negligible compared to the large difference in rETR between *Codium* slugs kept under low and high light (Fig. 2a,b). Furthermore, the rETR in *E*. *viridis* maintained on a *Codium* diet under different light regimes was positively correlated to the GE of the slugs (Fig. 1a versus Fig. 2a), indicating that the fitness benefit received by the slugs in high light was due to increased photosynthesis by functional kleptoplasts from the algal hosts.

**Table 1:** Repeated measures analysis of variance of the relative electron transport rate (rETR) of *Elysia viridis* consuming *Codium fragile* A) and *Cladophora rupestris* B) under low and high light (Light) during a four-week experiment (Time).

		*(A) E*. *viridis* on *Codium fragile* ^1^				*(B) E*. *viridis* on *Cladophora rupestris* ^2^			
	Variance component	df	MS	*F*	*P*	df	MS	*F*	*P*
**Between subjects**	Light	1	79239.46	**4475.51**	**< 0.01**	1	171.33	**34.90**	**< 0.01**
	Residual	38	17.71			38	4.91		
**Within subjects**	Time	2.55	266.38	**51.86**	**< 0.01**	2.68	5.24	0.53	0.65
	Time x Light	2.55	266.45	**51.87**	**< 0.01**	2.68	3.33	0.34	0.78
	Residual	96.79	5.14			101.78	9.97		

^1^G-G = 0.849, H-F = 0.939

^2^G-G = 0.893, H-F = 0.992

Values for the main factor Time and the Time x Light interaction are Greenhouse-Geisser adjusted as the assumption of sphericity of data was not met. Mean values and SEM are presented in Fig. 2.

**Fig 3:**
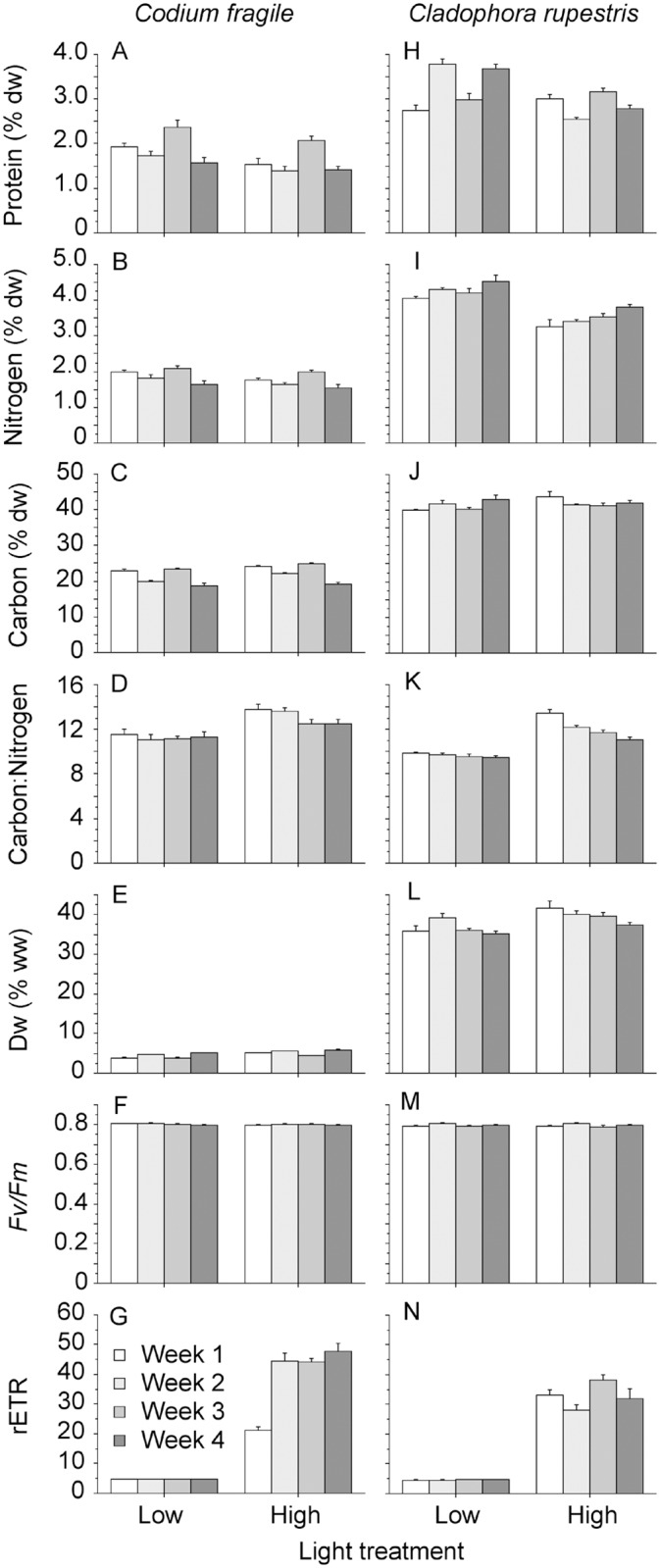
A and H) protein content (% dry weight), B and I) nitrogen content (% dry weight), C and J) carbon content (% dry weight), D and K) carbon:nitrogen ratio, E and L) dry weight (% wet weight), F and M) maximum quantum yield (*F*_v_/*F*_m_), and G and N) relative electron transport of photosystem II (rETR) of the two algal diets *Codium fragile* (A-G) and *Cladophora rupestris* (H-N) fed to *Elysia viridis* under low and high light during four-week experiments. Error bars show + SEM (n = 5).

There was significant temporal variation in almost all measured nutritional algal traits in both algal diet species during the four-week experiments (Table 2a,b). Furthermore, there were significant differences in most traits for *Codium* and *Cladophora* kept under low and high light (Table 2a,b). However, the differences between sampling occasions were mostly small, and there were no obvious temporal trends in any of the measured algal traits (Fig. 3a-n). The mean differences between light treatments were also small (< 3.2%) for all algal traits except for rETR (Fig. 3g,n). High *F*_v_/*F*_*m*_ for both algal species in high and low light treatments indicated that RCs in PSII were healthy in the algal diets provided to slugs (Fig. 3f,m). Moreover, rETR was much higher in high compared to low light treatments for both algal species (Fig. 3g,n).

**Table 2:** Analysis of variance of different traits of *Codium fragile* (*A*) and *Cladophora rupestris* (*B*) kept under low and high light (Light) during the four-week experiment (Time).

			(*A*) *Codium fragile*			(*B*) *Cladophora rupestris*		
	Variance component	df	MS	*F*	*P*	MS	*F*	*P*
**P (% dw)**	Light	1	0.94	**15.50**	**< 0.01**	1.78	**34.64**	**< 0.01**
	Time	3	1.09	**17.94**	**< 0.01**	0.23	**4.53**	**< 0.01**
	Light x Time	3	0.02	0.37	0.78	1.42	**27.51**	**< 0.01**
	Residual	32	0.06			0.05		
**C (% dw)**	Light	1	19.53	**19.33**	**< 0.01**	6.70	1.69	0.20
	Time	3	56.34	**55.75**	**< 0.01**	5.55	1.40	0.26
	Light x Time	3	1.14	1.13	0.35	10.03	2.53	0.08
	Residual	32	1.01					
**N (% dw)**	Light	1	0.23	**9.41**	**< 0.01**	6.05	**101.28**	**< 0.01**
	Time	3	0.37	**14.96**	**< 0.01**	0.45	**7.54**	**< 0.01**
	Light x Time	3	0.01	0.38	0.77	0.02	0.34	0.79
	Residual	32	0.02			0.06		
**C:N ratio**	Light	1	32.06	**34.54**	**< 0.01**	59.81	**297.87**	**< 0.01**
	Time	3	1.51	1.62	0.20	3.49	**17.37**	**< 0.01**
	Light x Time	3	1.13	1.21	0.32	1.83	**9.12**	**< 0.01**
	Residual	32	0.93			0.20		
**dw (% ww)**	Light	1	7.49	**107.77**	**< 0.01**	97.49	**17.70**	**< 0.01**
	Time	3	3.62	**52.15**	**< 0.01**	22.97	**4.17**	**0.01**
	Light x Time	3	0.14	1.98	0.14	11.09	2.01	0.13
	Residual	32	0.07			5.51		
***F***_**v**_ **/*F***_**m**_	Light	1	4.66×10^-5^	0.80	0.38	1.96×10^-5^	0.17	0.68
	Time	3	1.14×10^-4^	1.96	0.14	4.76×10^-4^	**4.16**	**0.01**
	Light x Time	3	5.36×10^-5^	0.92	0.44	1.61×10^-5^	0.14	0.94
	Residual	32	5.80×10^-5^			1.14×10^-4^		
**rETR**	Light	1	11994.93	**1052.79**	**< 0.01**	7932.50	**578.34**	**< 0.01**
	Time	3	372.25	**32.67**	**< 0.01**	44.85	**3.27**	**0.03**
	Light x Time	3	375.49	**32.96**	**< 0.01**	43.43	**3.17**	**0.04**
	Residual	32	11.39			13.72		

The traits measured include protein (P), carbon (C), nitrogen (N), carbon:nitrogen (C:N) ratio, dry weight (dw), maximum quantum yield (*F*_v_/*F*_m_), and the relative electron transport rate (rETR). Mean values and SEM are presented in Fig. 3.

*E*. *viridis* consuming *Codium* showed high values of *F*_v_/*F*_*m*_ and demonstrated similar rETR patterns to the *Codium* diets in each light treatment (Fig. 2a vs. 3g). In contrast, *E*. *viridis* consuming *Cladophora* had much lower *F*_v_/*F*_*m*_ and rETR regardless of light treatment compared to the *Cladophora* diets (Fig. 2b versus 3n). This indicates that while chloroplasts in *Cladophora* diets were healthy, they were not maintained in a functional state in *E*. *viridis*.

## Discussion

This work emphasizes the fitness advantages that a constant supply of photosynthetically functional kleptoplasts can provide mixotrophic hosts, and suggests that there may be substantial fitness trade-offs resulting from diet selection. Moreover, our study provides evidence that kleptoplasty can enhance trophic transfer efficiency via phototrophy by increasing the GE of a metazoan host, as has previously been demonstrated in kleptoplastic protists [3,41]. As predicted, *E*. *viridis* feeding on *Codium* demonstrated increased GE under high compared to low light, which correlated to increased rETR (i.e. photosynthesis) of the slugs, but not to any nutritional traits of the algal diet. In contrast, *E*. *viridis* feeding on *Cladophora* under high light did not display any increase in GE. This was likely due to the limited ability of *Cladophora* kleptoplasts to carry out photosynthesis in *E*. *viridis*, indicated by the low rETRs of slugs in both light regimes. Different light treatments induced statistically significant differences in some of the nutritional traits of both *Codium* and *Cladophora*, but the magnitudes of the differences were not considered large enough to cause the observed increase in GE in *Codium* slugs under high light. Furthermore, growth increases in other mesoherbivores (amphipods, isopods, and opisthobranch molluscs) of similar magnitudes as in the present study, required large changes in nutritional algal diet traits [42–44]. Together, these results imply that the differences in light-dependent GE for *E*. *viridis* feeding on *Codium* and *Cladophora* were due to variation in the kleptoplast functionality of the algal diets, rather than to their nutritional differences.

Kleptoplast/chloroplast functionality (*F*_v_/*F*_m_) and photosynthesis (rETR) assessed using PAM fluorometry were similar in *E*. *viridis* feeding on *Codium* and in the algal individuals used as diets, which concur with a previous study using similar techniques [32]. The photosynthetic ability of kleptoplasts in *E*. *viridis* decreases under starvation [13,32], but constant replenishment of functional kleptoplasts while feeding [45], may have explained the consistently high rETRs observed in *Codium* fed slugs under high light in the present study. In contrast, *F*_v_/*F*_m_ and rETR for *E*. *viridis* fed *Cladophora* were low and did not mirror values of the algal individuals used as food. These results indicate that, although the photosynthetic apparatus of *Cladophora* was intact during the experiment, *E*. *viridis* are unable to retain functional kleptoplasts from this diet. The reason for this could be that the chloroplasts of *Cladophora* are less “robust” compared to chloroplasts of *Codium*, and cannot withstand the physical stress associated with kleptoplasty [31, 46].

Previous attempts to assess light-dependent growth of *E*. *viridis* feeding on *Codium* presumably failed to show increased GE because slugs were kept in complete darkness instead of low light, which caused slugs to become lethargic [13]. *Codium* has a photosynthetic compensation point of 9.3 μmol quanta m^-2^ sec^-1^ [47], although positive growth has been documented in the *Codium* ssp. *tomentosoides* at 7 μmol quanta m^-2^ sec^-1^ [48]. We selected a light intensity that was low enough to substantially inhibit photosynthesis by *Codium* kleptoplasts, but in which *E*. *viridis* growth could occur [49]. Furthermore, we selected small individuals (< 25 mg) that were more likely to direct assimilated energy to somatic growth rather than reproduction. Assuming that low light conditions only supplied sufficient light for *Codium* kleptoplasts to overcome their own respiratory requirements, then growth in *E*. *viridis* under the low light regime could be considered as heterotrophy from grazing and any growth in excess of this value in high light attributable to phototrophy from kleptoplasts. A rough approximation, calculated using GEs from the specific light conditions, would attribute 53% of *E*. *viridis* growth to heterotrophy through grazing and 47% to phototrophy from *Codium* kleptoplasts under the high light treatment. This approximation is not unreasonable as estimates using stable carbon isotope ratios have indicated up to 60% of the total carbon input in sacoglossans may be from photosynthesis [50]. However, our approximation assumes that respiration rates and absorption efficiencies through grazing of *E*. *viridis* are equal under both light regimes, an assumption that may not be sound [51]. Furthermore, the light-dependent contributions of nitrogenous compounds assimilated by *Codium* kleptoplasts [26] to the GE of *E*. *viridis* are not known. Therefore, more accurate estimations of the light-dependant variation in carbon assimilated from grazing, and photosynthesis by kleptoplasts, versus carbon requirements of respiration, mucus production, and their relationship to *E*. *viridis* size and chlorophyll content should be conducted [52].

Our lab-based approach suggests there may be a substantial trade-off involved in diet selection attributable to functional kleptoplasts. However, the significance of kleptoplasty in an ecological setting may be very different. As algae represent both food and shelter to many marine mesoherbivores, including *E*. *viridis*, selection for algal diets that optimize fitness traits such as growth and reproduction may be outweighed by other factors such as susceptibility to predation [42,53] or removal from hosts by wave force [54]. In the study area, sympatric *E*. *viridis* are less abundant on *Codium* compared to *Cladophora* and individuals tend to be smaller on the former algal species compared to the latter [55]. This indicates that kleptoplasty may not be a significant factor in diet selection for *E*. *viridis* in the field. Increased GE presumably results in a shorter time to reach sexual maturity and/or greater reproductive output due to increased size [56,57]. Furthermore, gains in growth via phototrophy might offset the need for constant heterotrophic grazing, leading to a less rapid depletion of the algal host, reducing the need to constantly expend energy foraging for new sources of food [11]. However, larger size has its caveats, and for a cryptic mesoherbivore such as *E*. *viridis* increased size may have negative consequences through greater apparency to visual predators and susceptibility to removal by wave forces. In this respect, *Cladophora* might represent a superior host relative to *Codium*. Despite affording poor growth to *E*. *viridis* through grazing and limited photosynthesis through kleptoplasts, *Cladophora’s* filamentous morphology may provide better refuge from detection by visual predators, and its prostrate growth form may reduce exposure to the force of waves. This runs counter to *Codium*, which displays a simple planar morphology and grows upright in the water column. However, knowledge of algal host morphology and its role in circumventing biotic and abiotic pressures on *E*. *viridis* is limited, as is the role of predation pressure on the species.

In conclusion, using the natural variation in kleptoplast functionality in *E*. *viridis* feeding on different algal diets, we show that a combination of heterotrophy and phototropy can provide sacoglossan sea slugs with a significant increase in GE. Furthermore, we have uncovered a potentially large trade-off involving the selection of diets that do not afford functional kleptoplasts. However, we suggest that more accurate assessments of the relative contributions of heterotrophy and phototropy to growth in *E*. *viridis* are required, as are studies on the ecological consequences of kleptoplasty in natural slug populations in the field.

## Supporting Information

S1 Table Manuscript raw data. The file includes the raw data underlying the statistical analyses and figures in the manuscript.
(XLSX)
